# PHA Production from Cheese Whey and “Scotta”: Comparison between a Consortium and a Pure Culture of *Leuconostoc mesenteroides*

**DOI:** 10.3390/microorganisms9122426

**Published:** 2021-11-25

**Authors:** Francesca Bosco, Simona Cirrincione, Riccardo Carletto, Luca Marmo, Francesco Chiesa, Roberto Mazzoli, Enrica Pessione

**Affiliations:** 1 Department of Applied Science and Technology, Politecnico di Torino, 10129 Turin, Italy; luca.marmo@polito.it; 2 Structural and Functional Biochemistry, Laboratory of Microbial Biochemistry and Proteomics, Department of Life Sciences and Systems Biology, Università di Torino, 10123 Torino, Italy; simona.cirrincione@ispa.cnr.it (S.C.); roberto.mazzoli@unito.it (R.M.); enrica.pessione@unito.it (E.P.); 3 CNR-STIIMA, Consiglio Nazionale delle Ricerche- Istituto di Sistemi e Tecnologie Industriali Intelligenti per il Manifatturiero Avanzato, 13900 Biella, Italy; riccardoandrea.carletto@stiima.cnr.it; 4 Department of Veterinary Science (DSV), Università degli Studi di Torino, 10095 Grugliasco, Italy; francesco.chiesa@unito.it

**Keywords:** biodegradable bio-based polymers, byproduct valorization, lactic acid bacteria, fermentation, activated sludge consortia

## Abstract

It is urgent to expand the market of biodegradable alternatives to oil-derived plastics owing to (i) increasingly limited oil availability/accessibility, and (ii) the dramatic impact of traditional plastics on aquatic life, the food chain, all Earth ecosystems, and ultimately, human health. Polyhydroxyalkanoates (PHAs) are promising biodegradable polymers that can be obtained through microbial fermentation of agro-industrial byproducts, e.g., milk and cheese whey. Here, the PHA-accumulating efficiency of a mixed microbial culture (MMC, derived from activated sludges) grown on dairy byproducts (cheese and scotta whey) was measured. Bioreactor tests featuring temperature and pH control showed that both scotta and pre-treated Toma cheese whey could be used for PHA production by MMC, although scotta cheese whey supported higher PHA yield and productivity. The advantages of open MMCs include their plasticity and versatility to fast changing conditions; furthermore, no growth-medium sterilization is needed prior to fermentation. However, the use of pure cultures of efficient PHA producers may support better metabolic performances. Therefore, PHA-producing strains were isolated from a MMC, leading to the satisfactory identification of two bacterial strains, *Citrobacter freundii* and *Leuconostoc* spp., whose ability to accumulate PHAs in synthetic media was confirmed. A more detailed investigation by mass spectrometry revealed that the strain was *L. mesenteroides*. Although the validation of *L. mesenteroides* potential to produce PHA through fermentation of agro-industrial byproducts requires further investigations, this is the first study reporting PHA production with the *Leuconostoc* genus.

## 1. Introduction

Petrol-derived plastic polymers are recalcitrant to biodegradation and persist in natural environments. Approximately 50% of all plastics are bottles and packaging materials, which are improperly disposed and floating (an average of 270,000 tons) on the surface of the sea [1]. Through UV light exposure and other weathering processes, floating plastic wastes form microparticles and nanoparticles that can be ingested by marine turtles, large cetaceans, seabirds, fish, thus reaching human beings through the food chain [2]. These aspects, together with the increasing cost of oil extraction, have prompted researchers to investigate renewable polymers.

PHAs are high molecular weight (about 105 Da) polyesters, produced by bacterial anabolism, that exhibit several features similar to oil-derived plastics with the added value of biodegradability and biocompatibility [3,4]. These advantages result from the fact that the monomeric units of these polyesters are always in the R(-) configuration, due to the stereo-specificity of PHA synthases [5,6]. Therefore, many microorganisms can degrade PHAs using depolymerizing enzymes (e.g., *Pha*Z).

More than 150 naturally biosynthesized PHAs have been identified; however, chemical modification of naturally occurring PHAs [7], as well as the use of engineered bacteria [8] to produce higher performing PHAs, can account for a larger number of biopolymers. Among the different kinds of PHAs, the most interesting from a commercial viewpoint are poly(3-hydroxybutyrate) (P(3HB)) and poly(3-hydroxybutyrate)-co-(3-hydroxyvalerate) co-polymer (P(3HB-co-3HV)) whose physical and mechanical properties are very similar to those of traditional plastics [9].

Currently, PHAs are not competitive with oil-derived polymers because of their high cost [10]. However, the use of inexpensive carbon sources as PHA carbon feedstock, such as industrial and agriculture byproducts, can contribute to a reduction of about 40% of the overall production costs. PHA production using vegetable oils [11], swine wastewater [12], molasses [13], wheat and rice bran [14,15] and activated sludge wastewaters enriched with milk whey [16] has been proposed in the past two decades.

The huge amounts of carbon-rich milk whey make this industrial byproduct one of the most promising [17]. Milk whey is the watery portion after the precipitation of casein and fat from whole milk, and it is rich in lactose, lactoglobulins and lactoalbumins, minerals, and vitamins [18]. Part of the whey produced by the dairy sector is transformed in nutritional supplements or animal feed, while the rest is disposed as waste, causing environmental problems owing to its high biochemical oxygen demand (BOD, 40,000–60,000 ppm) and chemical oxygen demand (COD, 50,000–80,000 ppm) [19]. To increase lactose utilization, milk whey is generally pre-treated to decrease residual lipid and protein concentration [16]. A different way to exploit the caseification supernatant is to cook milk whey to produce ricotta. Ricotta is a low-fat casein-free food, which still contains albumins, globulins, and lactoferrin as its main proteins. Once ricotta has been separated, a supernatant enriched in salts and organic acids called scotta is obtained.

Due to all these considerations, the screening of natural bacteria able to grow on dairy byproducts has attracted and still attracts the attention of several research groups (see the extensive review by Amaro et al. [20]). The use of mixed microbial cultures (MMCs), although they have been associated with lower yields of PHA production, has two advantages: (i) MMCs do not require sterile conditions and (ii) they are able to adapt to changing industrial waste complex substrates [21,22]. In fact, studies on MMCs have shown that they consist of diverse bacterial genera, which change according to the tested fermentation carbon source [23]. A very promising approach is the use of open mixed cultures, such as those from activated sludges, since these bacteria intrinsically display plasticity and versatility to fast changing conditions [24,25]. To date, the use of pure cultures for efficient production of PHA from whey is challenging, because good PHA producers have displayed poor growth on lactose, whereas good lactose utilizers only direct a small part of their metabolism to PHA production [20]. Nevertheless, in spite of these considerations, a strain of *Alcaligenes latus* (Gram-negative) has been described as a good PHA producer when grown on whey [26]. It is evident that the use of pure cultures of lactic acid bacteria (LAB) could support better metabolic performances on whey because LAB have evolved in the milk ecological niche and are therefore well adapted to this environment [27]. However, their use for PHA production from whey has been poorly explored so far.

The present investigation was aimed to test cheese whey and scotta as carbon substrates for supporting growth and PHA accumulation of a microbial consortium originally isolated from activated sludges. Both biomass and PHA yields, along with the biochemical-metabolic characterization of the strains involved in the consortium, was determined. In parallel, a *Leuconostoc mesenteroides* strain was selected. It was grown as a pure culture and tested to compare its performances with those of the mixed microbial culture (MMC).

## 2. Materials and Methods

### 2.1. PHA Production with Mixed Microbial Cultures (MMC)

PHA production with MMC was performed in two different media: non-treated scotta whey derived from ricotta cheese production and pre-treated Toma cheese whey. In the latter case, thermo-calcic precipitation and an ultrafiltration process were applied in order to diminish lipid and protein content, as previously reported [28].

Scotta cheese whey tests were carried out both in Erlenmeyer flasks and in a 10 L bioreactor, while fermentation with Toma cheese whey was performed in a bioreactor. In all the tests (each one repeated three times), MMC inoculum was used; it was enriched from a dairy activated sludge plant in a synthetic medium containing acetic acid (20 g/L) as carbon source [21]. Cultures were incubated in the dark, at 30 °C and an initial pH of 7.0, under agitation on a rotary shaker at 120 rpm (bioSan PSU-20i, bioSan, Riga, Latvia). Biomass in the exponential growth phase (about 75 h) was collected and used as inoculum (10% *v*/*v*); the enrichment cultures were diluted to obtain an optical density at 620 nm (OD_620_) between 0.8 and 1 (Lambda 465 UV/Vis spectrophotometer (PerkinElmer, Waltham, MA, USA)).

#### 2.1.1. Flask Tests

Biomass in the exponential growth phase (about 75 h) was collected and used as inoculum for PHA production cultures. Enriched MMC (20 mL) was added to 180 mL of Scotta cheese whey in 500 mL Erlenmeyer flasks and incubated in the dark, at 30 °C, on a rotary shaker at 120 rpm. Cell growth was monitored by measuring OD_620_. Culture broth samples were collected at different times. Biomass was harvested by cenrifugation at 18,000 rpm and 4 °C for 10 min (MPW-380R, Warszawa, Poland) in pre-weighted glass tubes and dried to a constant weight at 60 °C for 48 h (Binder ED115, Tuttlingen, Germany).

#### 2.1.2. Bioreactor Tests

Batch fermentations were performed in a R’ALF Plus AG Bioreactor (Bioengineering AG, Wald, Switzerland) with an operative volume of 4 L. The temperature of the culture broth was maintained at 30 °C under agitation (120 rpm); pH was regulated at 7 and its regulation was obtained through acid (0.5 M H_2_SO_4_) and base (5 M NaOH) addition (both reagents were purchased at Sigma-Aldrich (St. Louis, MO, USA)). As for the flask tests, scotta whey derived from ricotta cheese production was used. Moreover, tests with pre-treated Toma cheese whey [28] were performed.

### 2.2. PHA Production with Leuconostoc mesenteroides Isolated from MMC

Batch fermentation tests with *L. mesenteroides* were performed in Erlenmeyer flasks using a modified Khardhenavis synthetic medium [21] in which acetic acid was replaced by glucose or lactose (40 g/L) as a carbon source. pH values were 6.4 and 6.5 for the media containing glucose and lactose, respectively. *L. mesenteroides* colonies picked up from a malt extract agar (MEA, 20 g/L malt extract, 2 g/L peptone, 20 g/L glucose, 20 g/L agar, all the reagents were purchased at Sigma-Aldrich (St. Louis, MO, USA)) plate were expanded in a De Man, Rogosa, Sharpe (MRS) [22] liquid medium (OXOID Ltd, Basingstoke, UK), in static conditions at 30 °C, for 24 h. Then, biomass derived from the 16 h pre-culture in MRS was used as inoculum (10% *v*/*v*). Culture conditions were the same as reported for the fermentation in Scotta cheese whey, described above.

Fermentation tests (each one repeated three times) were carried out for 120 h and samples were harvested at 24, 48, and 120 h to evaluate biomass dry weight and PHA production.

### 2.3. PHA Extraction

Biomass was pelletized by centrifugation (15,000 rpm, 10 min, 4 °C), in pre-weighted glass tubes and then dried at 60 °C for 48 h. The polymer was extracted from dried biomass according to the method of chloroform-hypochlorite dispersion described by [23].

PHA yield (*Y_PHA/X_*) was calculated at different fermentation times (*t*), as the ratio of PHA dry weight (g) and biomass dry weight (g), as follows:

$${\left(Y_{PHA/X}=\frac{\Delta c_{PHA}}{\Delta c_{biomass}}\right)}_{t}$$

$\Delta c_{PHA}$: difference between PHA concentration (g) at sampling times *t*_s_ and *t*_s-1_;

$\Delta c_{biomass}$: difference between biomass concentration (g) at sampling times *t*_s_ and *t*_s-1_.

The productivity (*P_PHA_*) was calculated at different fermentation times (*t*) as the volumetric rate of PHA generation as follows:

$${\left(P_{PHA}=\frac{\Delta c_{PHA}}{V\;\Delta t}\right)}_{t}$$

$\Delta c_{PHA}$: difference between PHA concentration (g) at sampling times *t*_s_ and *t*_s-1_;

V: fermentation volume (L);

Δt: difference between sampling times (h) *t*_s_ and *t*_s-1_.

### 2.4. Detection of PHA Producing Strains

MEA plates were inoculated with samples derived from Scotta flask cultures, harvested at different fermentation times (10 mL, every 24 h). Serial dilutions (10^−5^ or 10^−6^) were prepared using 0.9% NaCl solution; 100 µL of diluted samples were spread on MEA plates. After two to seven days of incubation at 30 °C, potential PHA producers were detected by Sudan Black B (SBB) staining of the colonies [29]. According to [30], a 0.002% SBB solution in 97% ethanol was gently spread over the plates, completely soaking them. They were incubated at room temperature for 30–60 min and then discarded and washed with 97% ethanol. PHA producers tend to be stained dark-blue or black, while negative PHA accumulators remains white or light blue. Counting of the different kind of colonies (white and coloured) was performed.

### 2.5. Isolation and Identification of PHA-Producing Bacteria

The PHA-producing bacteria detected by SBB staining and showing different morphology were isolated and plated as single colony in MRS and LB agar plates.

In order to identify these strains, they were Gram stained and subjected to microscope observation. Gram-positive coccoid cells were further analyzed by the API 20STREP test (bioMérieux SA, Marcy l’Etoile, France), while the API 20E and API 20NE (bioMérieux^®^) were used for Gram-negative rod cells according to the manufacturer’s instructions.

More detailed strain identification was determined by MALDI-TOF MS as previously described [31]. This method proved to have comparable reliability with 16S rRNA gene and *pheS* gene sequence analysis and provides important advantages over gene sequencing in terms of rapidity and cost per sample. The selected bacterial strain was loaded three times onto a steel MALDI target according to the manufacturer’s instructions (Bruker Daltonics, Bremen, Germany), overlaid with 1 μL of 70% formic acid, and dried at room temperature. Dried spots were then overlaid with 1 μL of HCCA (α-cyano-4-hydroxycinnamic acid) matrix solution in Bruker standard solvent (Sigma-Aldrich, Germany). After all of the samples had co-crystallized with matrix, the target plate was placed in the MALDI-TOF MS (Bruker Daltonics). The target was loaded into the Microflex LT bench-top mass spectrometer (Bruker Daltonics). The analysis was performed using FlexControl 3.4 software (Bruker Daltonics) and the MALDI bioTyper database (Compass Library MBT 8468 MSP, Bruker, Germany). An automatic measurement method was performed with the following parameters: mass range of 2000 to 20,000 Da, initial laser power at 25%, and maximal laser power at 35%. The calibration of the bacterial test standard was performed according to the manufacturer’s instructions. Species level was indicated for log score ≥ 2.0, genus level was indicated for log score < 2.0 and ≥1.7, and not reliable identification was indicated for log score < 1.7, as specified by the manufacturer.

## 3. Results

### 3.1. PHA Production with MMC

In order to evaluate the influence of medium composition on PHA production with MMC, the fermentation tests were performed with two different types of cheese whey: non-treated scotta cheese whey and pre-treated Toma cheese whey. The composition of the two different whey was reported in a previous work [28]. The main differences were in protein and lipid content, which was higher in Toma cheese whey (7.5 and 4 g/L, respectively) than in scotta (1.5 and 2 g/L, respectively). Lactose concentration was very similar, at 40 and 45 g/L in scotta and Toma cheese whey, respectively. In the fermentation tests, scotta was used as is, while Toma cheese whey was pre-treated in order to diminish the lipid and protein concentration.

#### 3.1.1. Flask Tests

**Figure 1:**
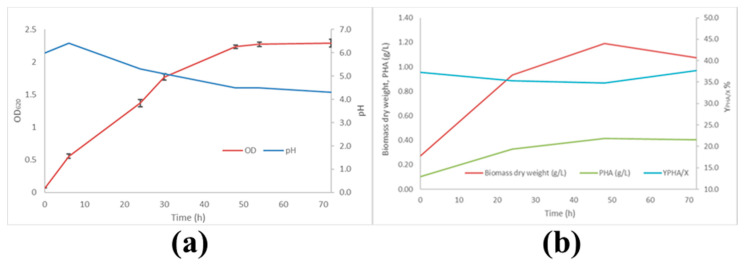
Growth and PHA production kinetics of MMC cultures in scotta cheese whey: (**a**) OD_620_ and pH; (**b**) biomass dry weight, PHA concentration, and PHA yield (data obtained from triplicate experiments).

The results obtained with scotta cheese whey are reported in Figure 1. In the flask tests, pH was measured, but not regulated.

In Figure 1a, the OD_620_ and pH profiles are shown. It is possible to observe an exponential growth phase up to 48 h, after which a stationary phase is evident. As far as the pH profile is concerned, a decrease from an initial value of 6.0 to 4.3 occurred in about 48 h. In fact, the pH curve presents an initial increase to 6.5, probably due to the consumption of acetic acid derived from the pre-culture medium. During the course of fermentation, biomass and PHA concentrations were measured at 24, 48, and 72 h. The obtained results, along with PHA yield, are reported in Figure 1b. It is possible to observe that microbial biomass reached the maximum concentration value (1.2 g/L) after 48 h, at the beginning of the stationary phase. Consistently, PHA concentration increased to a maximum (0.42 g/L) at 48 h, and then remained almost constant until the end of the fermentation. PHA yield, referring to the biomass (*Y_PHA/X_*), reached its maximum at 24 h of fermentation (35.73%) and then remained unchanged over time. In fact, the highest yield value registered at 72 h is due to the fact that the biomass concentration at this time was lower than that measured at 48 h, in spite of the constant concentration of the polymer. These results suggest that PHA is synthetized and accumulated mainly during the exponential growth phase.

**Figure 2:**
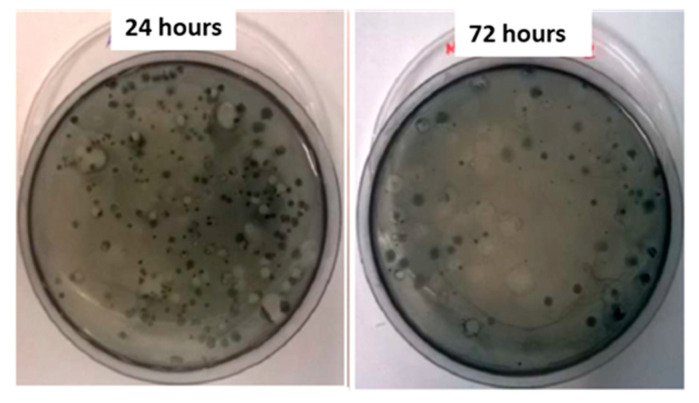
SBB staining of PHA producers at 24 h (**left**) and 72 h (**right**) of fermentation.

At different fermentation times, the percentage of positive colonies to SBB staining was also evaluated. The number of PHA producers was higher at 24 h (83%) than at the end of the fermentation course (64%) (Figure 2).

#### 3.1.2. Bioreactor Tests

Fermentation tests in a bioreactor were performed with both scotta and Toma cheese whey. In all the fermentation tests, temperature and pH values were regulated at 30 ± 0.2 °C and 7 ± 0.02, respectively. In Table 1, the maximum values of biomass, PHA concentration, PHA yield, and productivity were reported and compared with those obtained in flask tests. The relative standard error of the data reported is 5% (calculated from three replicates).

From the results obtained in scotta whey fermentation, the positive effect of pH control in the bioreactor was evident, as both the PHA yield and productivity were enhanced. In particular, the maximum value of biomass was almost doubled (1.2 in flask and 2.043 g/L in bioreactor) and PHA concentration was more than doubled (0.42 and 1.065 g/L) and reached earlier (29 h in bioreactor and 48 h in flasks).

Regarding bioreactor tests with Toma cheese whey, the maximum values of biomass (1.171 g/L) and PHA concentration (0.439 g/L) were obtained after 24 h of fermentation. Comparing the value of PHA yield (0.37 g/g), it is possible to observe that it was lower than that obtained in scotta bioreactor tests (0.52 g/g) and very similar to the value obtained in flask tests (0.35 g/g). Consequently, PHA productivity (0.018 g/L/h) was also lower in bioreactor tests.

### 3.2. Isolation and Identification of PHA-Producing Bacteria

Bacteria able to produce PHA were isolated from Scotta cheese whey on agar malt plates as colonies and stained by SBB solution. The viable count of PHA-producing colonies at three different times of fermentation showed a decrease in the PHA-producing bacteria along the fermentation kinetic. Precisely, 46.7%, 34.9%, and 23.4% of the total colonies were PHA-producers after 24, 48, and 72 h of fermentation, respectively.

Five different colonies were isolated according to their morphology (Table 2) after Gram staining and observation by optical microscopy. They consisted of four Gram-negative (two rods and two cocco-rods) and one Gram-positive (coccus) strains.

According to the morphology and Gram staining observed, the isolates were biochemically characterized by API tests. In particular, API 20E and API 20NE were used for Gram-negative colonies, in order to identify enteric and non-enteric species, while, API 20Strep was used for the Gram-positive colony.

The API test and the evaluation of the catalase activity allowed to obtain unambiguous results only for three isolates (colonies Number 2, 4, and 5). Two Gram-negative colonies (*n*. 2 and 5) were identified as the same bacterium, i.e., *Citrobacter freundii*, with a percentage of reliability of 99.9% (Table 2). The Gram-positive colony (Number 4) was identified as *Leuconostoc* spp., with a percentage of reliability of 98.5%. Unfortunately, the API results related to Colony 1 and Colony 3 showed doubtful identification. Two different bacteria were attributed to Colony 1 with low percentage of reliability, *Enterobacter cloacae* (56.4%) and *Raoultella ornithinolytica* (32.7%). Regarding Colony 3, both API 20E and API 20NE produced a robust result, *Escherichia coli* 96.3% and *Vibrio parahaemolyticus* 98.9% respectively, preventing a unique identification.

In order to obtain more detailed information on Colony 4, identified as *Leuconostoc* spp. at genus level only, a more in-depth evaluation of the API 20Strep results was performed. In particular, the positivity to glucosidase (ESC), indicating the ability to hydrolyze esculin, allowed the restriction of the identification to three species, *L. fallax*, *L. pseudomesenteroides*, and *L. mesenteroides* ssp. *mesenteroides* [32]. Eventually, MALDI-TOF MS determination led to the univocal identification of this strain as *L. mesenteroides* (Figure S1). The *Leuconostoc* genus includes obligate heterofermentative, mesophilic, and acidophilic bacteria showing an optimal growing temperature of 30 °C, which find applications in the dairy industry, even if they display a low growth rate (about 5–10% less than the other starters) [33]. Furthermore, *L. mesenteroides* is a GRAS organism; hence, it was selected for fermentation flask tests. This species and the whole *Leuconostoc* genus have never been described as PHA producers so far.

### 3.3. PHA Production with MMC-Isolated L. mesenteroides

The ability of the isolated strain to use glucose/lactose and produce PHA was tested in a synthetic medium. As reported in the Section 2, fermentation tests were performed in Khardhenavis medium with glucose or lactose as the only carbon source, at 30 °C.

**Figure 3:**
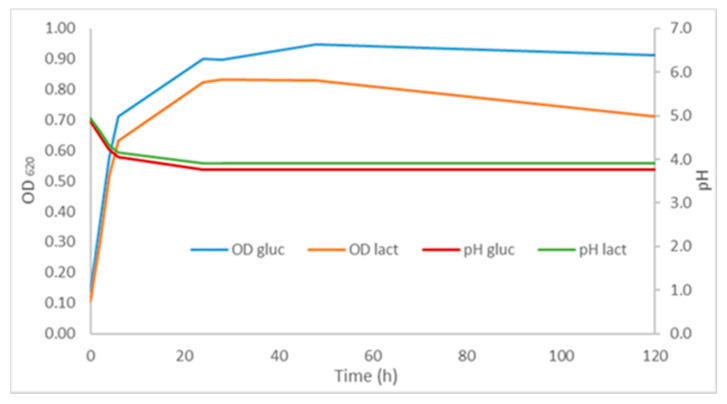
OD_620_ and pH kinetics of *L. mesenteroides.* grown on glucose or lactose-supplemented Khardhenavis synthetic media (data are obtained from triplicated experiments).

In Figure 3, the OD_620_ and pH profiles of these fermentation tests are reported. As shown, the growth curves in the two culture conditions were very similar. *L. mesenteroides* showed the ability to use both carbon sources at a high growth rate; nevertheless, biomass concentrations were very low compared with those obtained with MMC in the same synthetic medium (data not shown). The end of the exponential growth phase was probably due to the inhibitory effect of acidic pH (4.3), as reported in a previous study [34].

In Table 3, the main parameters of PHA production in flask tests have been reported, with a relative standard error of 5% (*n* = 3). Maximum PHA yield (36%) was the same in glucose and lactose fermentation tests; nevertheless, in the medium with glucose, the maximum was reached 24 h earlier. Consequently, the productivity values were double for medium with glucose (0.0035 g/L/h) with respect to that containing lactose (0.00119 g/L/h). These kinetics may be related to the time necessary for the bacterium to hydrolyze the disaccharide. These preliminary results confirm the ability of the isolated *L. mesenteroides* strain to produce PHA in a modified Khardhenavis synthetic medium containing glucose or lactose as a carbon source. However, the PHA yield and productivity of *L. mesenteroides* were lower than those obtained with MMC; therefore chemical-physical parameters (e.g., C and N concentrations, pH, agitation rate, and aeration) have to be optimized in culture flasks before bioreactor tests. These studies will be the subject of a future investigation.

In addition, further studies are necessary to confirm *L. mesenteroides* ability to accumulate PHA in scotta milk whey and the possible advantage of using pure *L. mesenteroides* with respect to MMC.

## 4. Discussion

The microbial world can provide a huge number of sustainable alternatives to petroleum-derived chemicals, including plastic polymers [35]. Several bacterial species can accumulate PHAs as carbon and energy storage material. High levels of PHAs can be obtained by *Methylobacterium organophilus* [36], *A. latus* [37], *Rhodopseudomonas palustris* [38], and especially *Cupriavidus necator* (originally called *Alcaligenes eutrophus* and later *Ralstonia eutropha*) [11]. Very recently, PHA production by halophilic archea has been proposed as well [39,40]. However, not all of these microorganisms are able to synthesize sufficient amounts of PHA for large-scale production [41]. PHA’s main metabolic role is as an energy reserve molecule, present in the cell as spherical inclusion bodies or granules [42]. Recently, PHAs’ additional functions in microbial physiology have also been reported, for example, their role in protecting bacteria from hydroxyl-radical attack [43,44,45]. Therefore, these molecules do not need to be abundant in the bacterial cell.

On the other hand, bio-based and biodegradable polymers constitute a real advantage only if they can be produced from renewable resources, including industrial byproducts and not food-competing sources [46,47]. *C. necator* is the most studied PHA producing model because of its ability to synthesize large amounts of P(3HB) from simple carbon substrates such as glucose, lactic acid, acetic acid, and P(3HB-co-3HV) from n-alkanoates [48]. Nevertheless, it is unable to metabolize more complex low-cost substrates such as molasses, starchy wastes, or whey. For this reason, mixed cultures of lactic acid producing bacteria such as *Lactococcus lactis* [49] or *Lactobacillus delbrueckii* [50] and *C. necator* have been used to bypass this bottleneck. The microbial ability to use lactose as a low cost carbon source is strictly dependent on the presence of β-galactosidase, a glycoside hydrolase enzyme that catalyzes the hydrolysis of lactose into its monosaccharide components, glucose, and galactose through the breaking of a glycosidic bond [17]. A recent review [20] showed that several microorganisms are able to convert whey lactose into PHA with productivity varying from 0.0035 g/L/h for MMC culture to 5.2 g/L/h for engineered *E. coli* strain. In the work of Raho et al. [51], a multi-step fractionation was used to recover a RCEW fraction containing 12.6% (*w*/*v*) of lactose that was enzymatically hydrolyzed. PHA yields obtained with *Haloferax mediterranei* in bioreactor tests were in the range 7–10 % (*w*/*w*), lower than those obtained in the present work (52% *w*/*w*).

The work of Berwig et al. [26] showed that *A. latus* is able to convert whey lactose into PHA with a productivity of 0.11 g/L/h. Although the yields are not comparable to those obtained in conventional media, discovering new bacterial species that can directly produce high levels of PHA from lactose is a promising step to obtaining economically competitive production of PHAs from whey. On the other hand, using an unknown mixed microbial community to produce PHAs can raise questions regarding biocompatibility when PHAs are to be used in medical applications. Furthermore, approaches exploiting wild-type bacteria can also circumvent the use of genetically engineered strains that requires more controlled production plants.

In the present study, a microbial consortium derived from an activated sludge plant was investigated for its ability to grow on scotta and pre-treated Toma cheese whey and to produce satisfactory amounts of PHAs. Both byproducts supported PHA production by MMC. However, the best result in term of PHA yield (0.52 g/g) and productivity (0.037 g/L/h) was obtained in bioreactor fermentation with scotta whey medium at a controlled pH value. Scotta can be considered as a complete medium for PHA production, without the need for any addition of other salts or substances. The application of MMC in PHA production could represent an advantage from an economic point of view, since sterility is not necessary. Moreover, the use of MMCs represents an interesting option for identifying new PHA producing microorganisms, as described in the previous paragraph [20].

We found that one of the most active and abundant PHA-producing bacterial population was *L. mesenteroides*. Since the microbial consortium was grown in scotta, the real origin of this bacterium is questionable. From one side we can speculate that this strain belongs to the rich multispecies microbiota present in the activated sludge. However, it is also reasonable to assume that it can belong to the scotta microflora, because no sterilization treatment was performed on it prior to fermentation, since when a mixed microbial population is used no selective condition is needed. The viability of *Leuconostoc* sp. in non-dairy environments such as activated sludges has been described by Lee et al. [52], along with its attitude to colonizing the milk ecosystem [53]. The most probable hypothesis is that the dairy environment of scotta has promoted a positive selection on the *Leuconostoc* population among the other non-lactic microorganisms. Moreover, possible syntrophic events occurring between activated sludge microbiota and scotta microbial population may have supported improved performances. In the present study, pure cultures of *L. mesenteroides* in synthetic media containing glucose or lactose as a carbon/energy source demonstrated that, in spite of the low cell number, good PHA yield was produced. In glucose-supplemented medium (C/N ratio about 107), the average PHA concentration was 0.09 g/L, with an average yield of 48%. In lactose-supplemented medium (C/N ratio about 112) the average PHA concentration was 0.06 g/L, with an average yield of 38%. This result is consistent with the higher energy expenditure required for galactose (in case of extracellular lactose hydrolysis occurs) or lactose (if hydrolysis occurs inside the cell) internalization and processing, including either permease-based proton symport systems or antiport systems involving galactose extrusion [54]. In the latter case, only half of the carbon substrate is available for intracellular metabolism. Furthermore, the synthesis of the beta-galactosidase enzyme also requires energy. Finally, the original ecological niche in which *L. mesenteroides* was selected (activated sludges) suggests that lactose cannot be the preferred sugar substrate for this strain.

Despite the high growth rates observed in both glucose and lactose-supplemented media, *L. mesenteroides* displayed poor biomass yield. This probably occurred because of a too high medium acidification, owing to the relative sensitivity of this bacterial genus to acidic pH. Actually, being an obligate heterofermenter, *L. mesenteroides,* besides producing lactic acid, also converts part of the monosaccharide substrate into gluconic acid and, in this case, acetic acid, which can lower the pH far below the natural tolerance of this genus (optimal pH around 5.5–5.8) [33]. A low tolerance of this genus to pH lower than 4.3 was previously reported [34]. However, in this study the real cellular damage hindering growth only occurred below pH 3.8. Furthermore, this problem can be easily overcome by performing cultures with regulated pH or by using complex media that support other elements, possibly buffering the environment. This is the case of scotta and whey, media rich in minerals and vitamins that exactly respond to this requirement.

To date, to the best of our knowledge, PHA synthesis in *L. mesenteroides* has never been reported. It would be interesting to outline the possible biosynthetic pathway for PHA production in this strain, but any effort in this direction is merely speculative, and out of the scope of the present investigation.

Considering that the building blocks for PHA generation are common metabolites (i.e., acetate and 2-oxobutyrate) available in almost all cellular systems, *L. mesenteroides* might have acquired the capability to produce esters from these very simple compounds by horizontal gene transfer. Recombination events, promoting enhanced metabolic and biosynthetic capabilities, are especially frequent in both the habitat of the activated sludge and in the whey ecological niche, where mixed and diversified bacterial populations exist. A possible strategy to better elucidate the biosynthetic route for PHA production in this strain is to compare protein expression profiles in control conditions and during PHA synthesis, by using intracellular gel-free proteomics. This approach will give reliable results and, therefore, deserves further investigations in the future to improve knowledge about *L. mesenteroides* biology and biochemistry.

## 5. Conclusions

This investigation proved that PHA production can be obtained by direct fermentation of dairy byproducts (scotta and pre-treated Toma cheese whey) by means of open microbial mixed cultures (MMCs). Within this context, scotta cheese whey supported higher PHA yield and productivity. A *L. mesenteroides* isolated from this MMC proved to be one of the bacterial strains contributing to PHA production in the MMC; therefore, this study is the first report in which a pure culture of *L. mesenteroides* was found able to produce PHA (with a yield of about 36%). Although further confirmation is necessary to assess the potential of isolated *L. mesenteroides* for utilization in production of PHA by fermentation of agro-industrial waste, these results suggest that the bioconversion of dairy byproducts into bio-based biopolymers by using lactic acid bacteria is a promising field worthy of additional investigation.

## Figures and Tables

**Table 1:** Main parameters of PHA production in flask and bioreactor tests with scotta and Toma cheese whey. The relative standard error of the data reported is 5% (calculated from three replicates).

Fermentation Mode	Medium	Time (h)	Biomass Dry Weight (g/L)	PHA (g/L)	*Y_PHA/X_* (g/g)	PHA Productivity (g/L/h)
Flask	Scotta cheese whey	48	1.2	0.42	0.35	0.0088
Bioreactor	Scotta cheese whey	29	2.043	1.065	0.52	0.037
	Toma cheese whey	24	1.171	0.439	0.37	0.018

**Table 2:** Identification of the PHA-producing bacteria isolated in this study by Gram staining and API test.

Colony	Gram Staining	Morphology	API Test	Catalase	API Result	Identification
1	Negative	Cocco-rods	E/NE	Positive	*Enterobacter cloacae* 56.4% (E) *Raoultella ornithinolytica* 32.7% (E)	Not acceptable
2	Negative	Rods	E/NE	Negative	*Citrobacter freundii* 99.9%	Acceptable
3	Negative	Cocco-rods	E/NE	Positive	*Escherichia coli* 96.3% (E) *Vibrio parahaemoliticus* 98.9% (NE)	Not acceptable
4	Positive	Coccus	STREP	Negative	*Leuconostoc* spp. 98.5%	Acceptable
5	Negative	Rods	E/NE	Negative	*Citrobacter freundii* 99.9%	Acceptable

**Table 3:** Main parameters of PHA production in flask test with *L. mesenteroides.* The relative standard error of the data reported is 5% (obtained from triplicated experiments).

Medium	Time (h)	Biomass Dry Weight (g/L)	PHA (g/L)	*Y_PHA/X_* (g/g)	PHA Productivity (g/L/h)
Modified Khardhenavis with glucose	24	0.2350	0.0850	0.036	0.0035
Modified Khardhenavis with lactose	48	0.1565	0.0570	0.036	0.00119

## Data Availability

Not applicable.

## Supplementary Materials

The following are available online at https://www.mdpi.com/article/10.3390/microorganisms9122426/s1, Figure S1: Report of the identification of *Leuconostoc mesenteroides* obtained by means of MALDI Biotyper.
