# Supplementary material for: PHA Production from Cheese Whey and “Scotta”: Comparison between a Consortium and a Pure Culture of Leuconostoc mesenteroides
Source: Microorganisms. 2021 Nov 25;9(12):2426. doi: 10.3390/microorganisms9122426 (PMC8704080; doi:10.3390/microorganisms9122426)
Supplement: Supplementary file 1 [file microorganisms-09-02426-s001.zip › microorganisms-1485225-supplementary.pdf]

Supplementary Material

PHA Production from Cheese Whey and "Scotta": Comparison between a Consortium and a Pure Culture of *Leuconostoc mesenteroides*

Francesca Bosco [1,*], Simona Cirrincione [2], Riccardo Carletto [3], Luca Marmo [1], Francesco Chiesa [4], Roberto Mazzoli [2] and Enrica Pessione [2]

[1] Department of Applied Science and Technology, Politecnico di Torino, 10129 Turin, Italy; francesca.bosco@polito.it (F.B.); luca.marmo@polito.it (L.M.)

[2] Structural and Functional Biochemistry, Laboratory of Microbial Biochemistry and Proteomics, Department of Life Sciences and Systems Biology, Università di Torino, 10123 Torino, Italy; simona.cirrincione@ispa.cnr.it (S.C.); roberto.mazzoli@unito.it (R.M.); enrica.pessione@unito.it (E.P.)

[3] CNR-STIIMA, Consiglio Nazionale delle Ricerche- Istituto di Sistemi e Tecnologie Industriali Intelligenti per il Manifatturiero Avanzato, 13900 Biella, Italy; riccardoandrea.carletto@stiima.cnr.it (R.C.)

[4] Department of Veterinary Science (DSV), Università degli Studi di Torino, 10095, Grugliasco-TO, Italy; francesco.chiesa@unito.it (F.C.)

* Correspondence: francesca.bosco@polito.it; Tel: +39-011-0904696

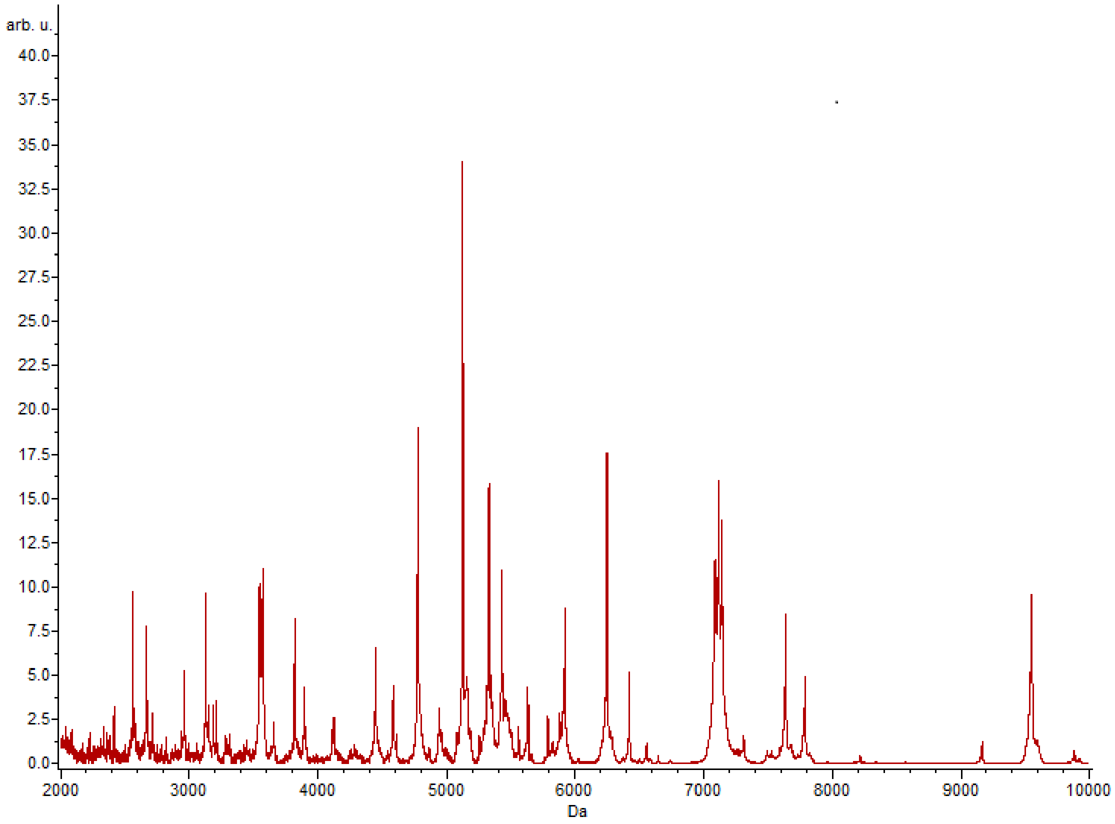

Figure S1. Report of the identification of *Leuconostoc mesenteroides* obtained by means of MALDI Biotyper.
